# Supplementary material for: Remodelled cholesteryl ester enriched lipid droplets fuel flavivirus morphogenesis
Source: bioRxiv. 2025 Sep 30:2025.06.07.658178. Preprint. [Version 3] doi: 10.1101/2025.0.607.658178 (PMC12645520; doi:10.1101/2025.0.607.658178)
Supplement: Supplement 1 [file media-1.pdf]

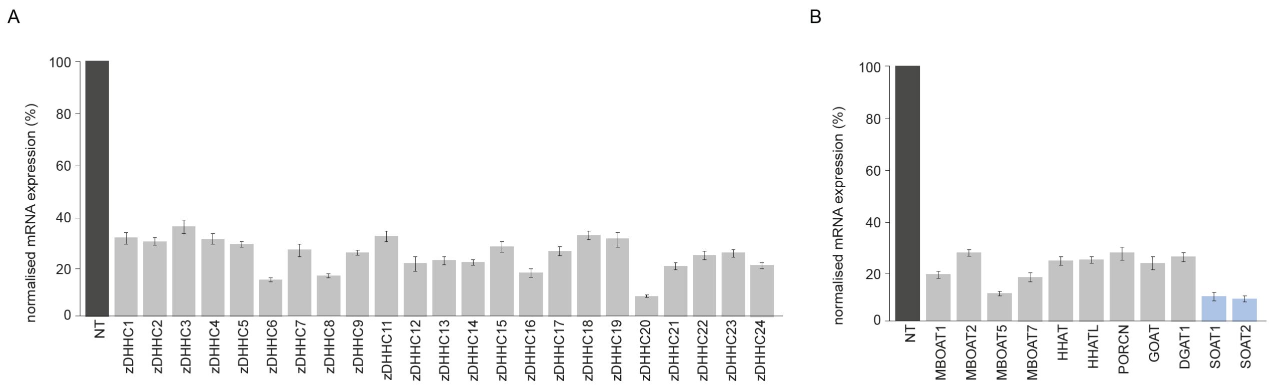

Figure S1: siRNA screen of zDHHC and MBOAT acyltransferases

(A) Normalised mRNA expression levels of zDHHC family genes following siRNA-mediated knockdown in Huh7 cells. NT, non-targeting control siRNA. **(B)** Normalised mRNA expression levels of MBOAT family genes following siRNA-mediated knockdown in Huh7 cells. Data are presented as mean ± SEM from three independent experiments. mRNA levels were quantified by RT-qPCR and normalised to the non-targeting control (set to 100%).

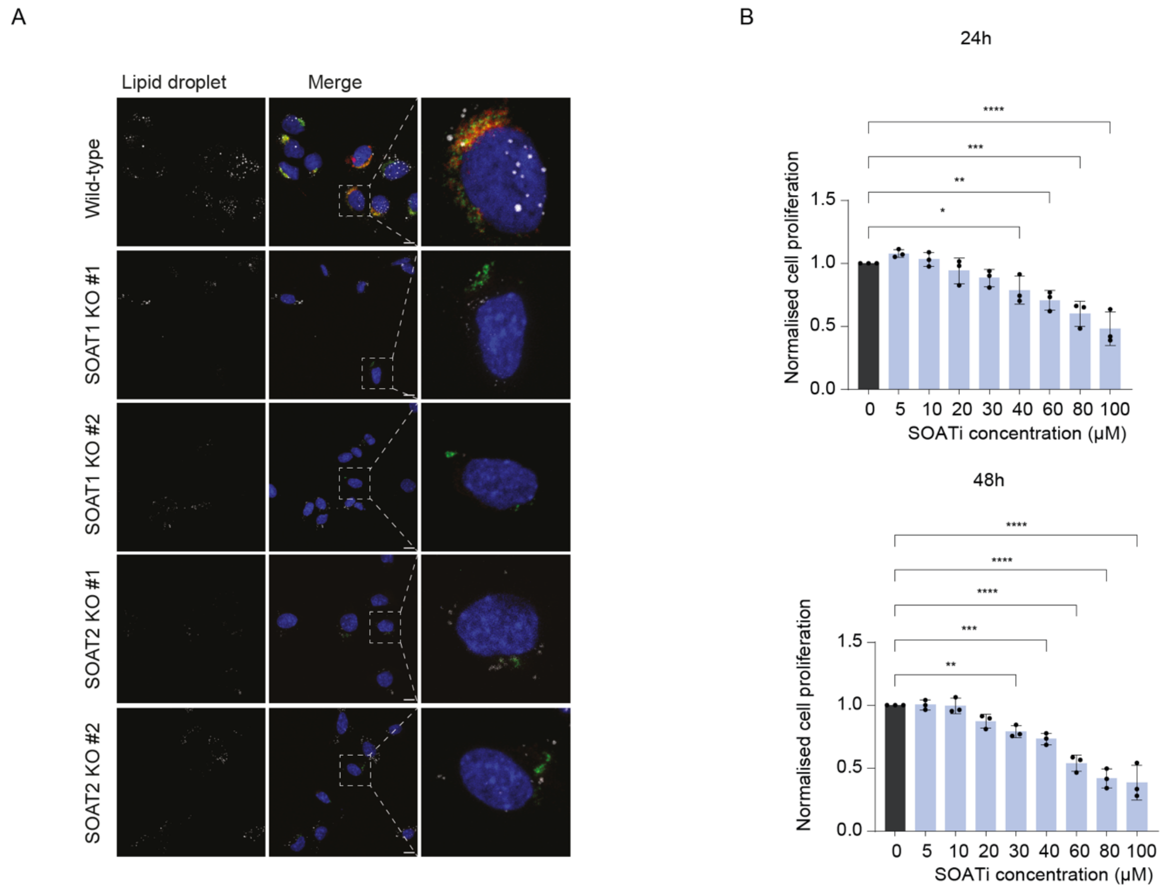

Figure S2: SOAT deficiency reduces lipid droplet abundance

(A) Confocal microscopy images of lipid droplets in wild-type and SOAT1/2 knockout cell lines. Lipid droplets were stained with BODIPY 493/503 (white), nuclei with DAPI (blue). Scale bar = 10 µm. Dashed boxes indicate regions shown at higher magnification in the right panels. Lipid droplet numbers are reduced in SOAT1 and SOAT2 knockout cells compared to wild-type cells. **(B)** Cell proliferation assay showing dose-dependent effects of SOAT inhibitor (SOATi) treatment at 24h and 48h. Huh7 cells were treated with increasing concentrations of Avasimibe (0-100 µM) and cell proliferation was measured. Data are presented as mean ± SEM from three independent experiments, normalised to untreated control. Statistical significance was determined by one-way ANOVA with Dunnett's multiple comparisons test (*p<0.05, **p<0.01, ***p<0.001, ****p<0.0001).

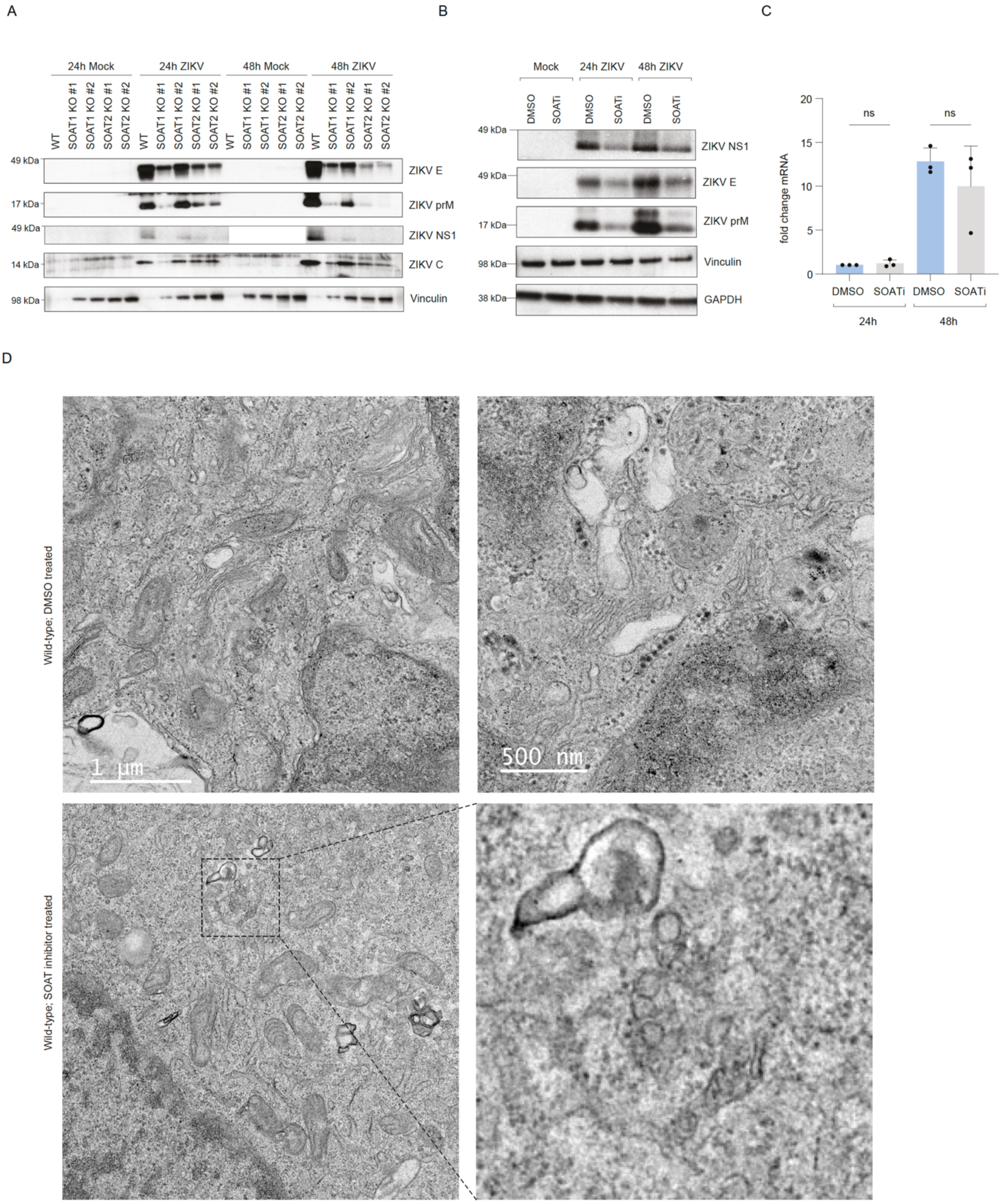

Figure S3: SOAT deficiency reduces viral protein levels and impair replication organelle formation.

(A) Western blot analysis of viral proteins in wild-type, SOAT1$^{-/-}$, and SOAT2$^{-/-}$ cells at 24 and 48 hours post-infection with ZIKV. Vinculin serves as loading control. Viral proteins (E, prM, NS1, capsid) are reduced in SOAT-deficient cells compared to wild-type. **(B)** Western blot analysis of viral proteins in wild-type cells treated with DMSO (control) or SOAT inhibitor (SOATi) at 24 and 48 hours post-ZIKV infection. GAPDH and Vinculin serve as loading controls. **(C)** Quantitative RT-

PCR analysis of viral RNA levels in DMSO- versus SOATi-treated cells at 24 and 48 hours post-infection. Data are presented as fold change relative to DMSO control (mean ± SEM, n=3 independent experiments). ns, not significant. **(D)** Transmission electron microscopy images of wild-type cells treated with DMSO (top panels) or SOAT inhibitor (bottom panels) at 24 hours post-ZIKV infection. Right panels show higher magnification views.
